# Pharmacological Targeting of BMP6-SMAD Mediated Hepcidin Expression Does Not Improve the Outcome of Systemic Infections With Intra-Or Extracellular Gram-Negative Bacteria in Mice

**DOI:** 10.3389/fcimb.2021.705087

**Published:** 2021-07-23

**Authors:** Alexander Hoffmann, Lara Valente de Souza, Markus Seifert, Laura von Raffay, David Haschka, Philipp Grubwieser, Manuel Grander, Anna-Maria Mitterstiller, Manfred Nairz, Maura Poli, Günter Weiss

**Affiliations:** ^1^ Department of Internal Medicine II, Infectious Diseases, Immunology, Rheumatology, Medical University of Innsbruck, Innsbruck, Austria; ^2^ Christian Doppler Laboratory for Iron Metabolism and Anemia Research, Medical University of Innsbruck, Innsbruck, Austria; ^3^ Department of Molecular and Translational Medicine, University of Brescia, Brescia, Italy

**Keywords:** hepcidin inhibitor, infection, Gram-negative bacteria, over-sulfated heparins, LDN-193189, *Salmonella*, *E. coli*, iron

## Abstract

**Introduction:**

Hepcidin is the systemic master regulator of iron metabolism as it degrades the cellular iron exporter ferroportin. In bacterial infections, hepcidin is upregulated to limit circulating iron for pathogens, thereby increasing iron retention in macrophages. This mechanism withholds iron from extracellular bacteria but could be of disadvantage in infections with intracellular bacteria. We aimed to understand the role of hepcidin in infections with intra- or extracellular bacteria using different hepcidin inhibitors.

**Methods:**

For the experiments LDN-193189 and oversulfated heparins were used, which interact with the BMP6-SMAD pathway thereby inhibiting hepcidin expression. We infected male C57BL/6N mice with either the intracellular bacterium *Salmonella* Typhimurium or the extracellular bacterium *Escherichia coli* and treated these mice with the different hepcidin inhibitors.

**Results:**

Both inhibitors effectively reduced hepcidin levels *in vitro* under steady state conditions and upon stimulation with the inflammatory signals interleukin-6 or lipopolysaccharide. The inhibitors also reduced hepcidin levels and increased circulating iron concentration in uninfected mice. However, both compounds failed to decrease liver- and circulating hepcidin levels in infected mice and did not affect ferroportin expression in the spleen or impact on serum iron levels. Accordingly, both BMP-SMAD signaling inhibitors did not influence bacterial numbers in different organs in the course of *E.coli* or S.Tm sepsis.

**Conclusion:**

These data indicate that targeting the BMP receptor or the BMP-SMAD pathway is not sufficient to suppress hepcidin expression in the course of infection with both intra- or extracellular bacteria. This suggests that upon pharmacological inhibition of the central SMAD-BMP pathways during infection, other signaling cascades are compensatorily induced to ensure sufficient hepcidin formation and iron restriction to circulating microbes.

## Introduction

Bacterial infections have a large impact on public health. In 2017, an estimated 48.9 million cases of sepsis and 11 million sepsis-related deaths were reported worldwide (Rudd et al., 2020). Bacterial infections are the most common cause of sepsis, and the severity can be partly linked to iron homeostasis as iron is an essential growth factor for many bacteria but also impacts on immune control (Ganz and Nemeth, 2015; Skaar and Raffatellu, 2015; Soares and Weiss, 2015; Brandtner et al., 2020; Valente de Souza et al., 2020). The successful withdrawal of iron by the host results in an improved infection outcome, a process called nutritional immunity (Weinberg, 1975; Skaar and Raffatellu, 2015; Gerner et al., 2020; Nairz and Weiss, 2020). The underlying mechanisms include modifications of systemic iron homeostasis as well as cellular iron trafficking by inflammatory or infectious stimuli. Specifically macrophages are in the center of cellular iron disturbances in the course of inflammation as they can acquire iron *via* multiple pathways including transferrin receptor mediated iron uptake, molecular iron incorporation *via* divalent metal transporter 1 (DMT1), and the solute carrier family 39 (zinc transporter) member 14 (Zip14) (Hentze et al., 2010), *via* hemopexin or haptoglobin receptors (Hvidberg et al., 2005; Schaer et al., 2006), or *via* ingestion of senescent or damaged erythrocytes (Nairz et al., 2017). These pathways are differently affected by cytokines or bacterial products thereby causing increased iron incorporation into macrophages (Byrd and Horwitz, 1993; Weiss et al., 1997; Mulero and Brock, 1999; Ludwiczek et al., 2003). In contrast, there is only one major iron export route from cells *via* the transmembrane exporter ferroportin (FPN1) (Donovan et al., 2000; McKie et al., 2000). This protein is also regulated by cytokines, radicals, or bacterial products which can determine the amount of iron being exported (Ludwiczek et al., 2003; Recalcati et al., 2010; Cherayil, 2011; Nairz et al., 2013). However, the most important regulatory effect on cellular iron export is mediated by the hepatic hormone hepcidin (HAMP), which binds to FPN1 subsequently leading to its occlusion or internalization and lysosomal degradation (Nemeth et al., 2004; Aschemeyer et al., 2018). Hepcidin expression is induced by multiple factors, including inflammatory stimuli resulting in reduced expression of FPN1 on duodenal enterocytes and on macrophages, thereby reducing either duodenal iron absorption or iron export from macrophages which reutilize iron from senescent erythrocytes (Nemeth et al., 2004; Theurl et al., 2009). Accordingly, inflammation inducible formation of hepcidin has been shown to cause hypoferremia and to exert benefit towards the course of infection with extracellular bacteria as it limits their access to iron (Arezes et al., 2015). Along that line, hepcidin supplementation could protect mice from lethal sepsis with the extracellular bacterium Escherichia coli (Stefanova et al., 2018). On the other site of the coin, hepcidin may be detrimental for infection with pathogens residing within cells such as macrophages as it increased their access to iron by reducing FPN1 expression (Chlosta et al., 2006; Drakesmith and Prentice, 2012; Nairz et al., 2013; Lokken et al., 2014). To systemically study the effect of hepcidin we used two mouse models of systemic infection with intracellular (S. typhimurium) and extracellular bacteria (E. coli) and investigated the course of infection upon application of two inhibitors of hepcidin formation.

Based on the cause- effective roles of high hepcidin levels in multiple diseases including anemia of inflammation or renal anemia (Macdougall, 2012; Nakanishi et al., 2015; Weiss et al., 2019), hepcidin inhibitors were developed for potential clinical use and analyzed in several pre-clinical models (Sun et al., 2012; Poli et al., 2014b; Petzer et al., 2018; Pagani et al., 2019).

The transcription of *Hamp* is positively regulated by different stimuli including iron, interleukin-6 (IL6) or LPS. Iron depending signaling involves the binding of BMP6 to its receptor (ALK2), which leads to the phosphorylation of SMAD1/5/8 that forms a complex with SMAD4, translocates to the nucleus where it binds to the SMAD responsive element (SMAD-RE) to activate *Hamp* expression (Hentze et al., 2010). The inflammatory pathway is driven by IL6 that binds to its receptor and activates the janus kinase 2 (JAK2). JAK2 then phosphorylates STAT3 which translocates to the nucleus, binds to the STAT3 responsive element (STAT3-RE) and activates *Hamp* gene expression (Hentze et al., 2010). Additionally, a LPS driven pathway exists, where LPS binds to the toll like receptor 4 (TLR4) and activates *Hamp* expression *via* myeloid differentiation primary response 88 (MyD88) (Lee et al., 2017).

Multiple approaches including neutralization of circulating hepcidin by different means or inhibition of hepcidin formation have been applied. Hepcidin inhibitors improved inflammation driven alterations of iron homeostasis or ameliorated anemia in animal models but also in humans, mainly by mobilization of iron from macrophages (Sasu et al., 2010; Theurl et al., 2011b; Boyce et al., 2016; Khorramian et al., 2017; Petzer et al., 2020).

However, until now, no data were available on the effects of hepcidin inhibitors in bacterial infections. We investigated two approaches of hepcidin inhibition. First, we used over-sulfated heparins (osH), in which the anticoagulant effect of heparin is no longer present due to the inhibition of binding to antithrombin by sulfation (Poli et al., 2014a). These osH are proposed to inhibit *Hamp* expression by interacting with bone morphogenic protein (BMP)/SMA and mothers against decapentaplegic (SMAD) signaling pathways. However, they do not affect another inflammation driven signaling pathway or hepcidin induction by inflammatory stimuli including IL6 *via* the signal transducer and activator of transcription – 3 (STAT3) pathway (Poli et al., 2014a; Girelli et al., 2016). The mechanism underlying the inhibition of Hamp by heparins is based on binding of heparins to BMPs, thereby blocking their interaction with the BMP type I activin receptor like kinase 2/3 (ALK2/3) (Billings et al., 2018; Denardo et al., 2021).

The second inhibitor used for this study is the dorsomorphin analogue, LDN-193189 (LDN), which efficiently suppresses the signaling of ALK2/3/6 and therefore *Hamp* expression (Yu et al., 2008). LDN was shown to effectively suppress *Hamp* expression in healthy mice and chronically inflamed rats thereby ameliorating anemia (Steinbicker et al., 2011b; Theurl et al., 2011b).

We used two models of systemic bacterial infection: *Salmonella enterica* serovar Typhimurium (S.Tm) is a Gram-negative facultative intracellular bacterium that can cause typhoid like fever in mice. Upon infection, *Salmonella* invades a habitat called the *Salmonella*-containing vacuole (SCV) within macrophages where this bacterium can persist depending on the efficacy of immune response and its supply to nutrients including iron (Andrews-Polymenis et al., 2010).

*Escherichia coli* (E. coli) is an extracellular Gram-negative bacterium, which is the most common cause of urinary tract infections but can also be found in up to 60% of all cases of acute bacterial peritonitis. Like almost all pathogens, both bacteria are dependent on a sufficient supply of iron for their growth and virulence (Malik-Kale et al., 2011; Yu et al., 2019).

## Materials and Methods

### *In Vitro* Experiment

FL83B immortalized hepatocytes from C57/BL6 mice (Breslow et al., 1973) were seeded in 6- or 12-well plates using Williams E media (Gibco) with 10% FBS (PAN-Biotech), 1% Penicillin/Streptomycin (Lonza) and 1% Glutamin (Lonza). As hepcidin inhibitors, we used LDN-193189 hydrochloride (Axon Medchem), and as a vehicle 2% (wt/vol) (2-Hydroxypropyl)-β-cyclodextrin (Med Chem Express) in PBS (Lonza) as well as oversulfated heparins, provided by Ronzoni Institute (Milan, Italy), dissolved in PBS as a vehicle. As stimulants, we used lipopolysaccharide (LPS) from *Salmonella* Typhimurium (Sigma) and recombinant mouse IL6 (eBioscience). For *in vitro* experiments we treated the cells with either 500nM LDN or with 3.6µg/ml of osH for 6h, as those concentrations of the inhibitors were already used previously (Theurl et al., 2011b; Poli et al., 2014a). To mimic an inflammatory situation, we stimulated cells with either 10 ng/ml of LPS or 100 ng/ml IL6 together with osH or LDN for 6h.

### *In Vivo* Experiment

C57Bl/6N (Charles River) mice were kept under a constant light/dark cycle on a standard diet (Ssniff) and had access to food and water *ad libitum*. Male mice at the age of 10 weeks received either 3 mg/kg-bodyweight of LDN-193189 dissolved in 200 µl of 2% (2-Hydroxypropyl)-β-cyclodextrin intraperitoneally (i.p.) or only the solvent (vehicle) 2% (2-Hydroxypropyl)-β-cyclodextrin alone i.p. 1h prior to the infection and a second dose 11h after the infection. For the inhibition with oversulfated heparins, mice received 40 mg/kg-bodyweight of oversulfated heparins dissolved in 100 µl PBS subcutaneously or an equal volume of PBS alone 1h prior to the infection and a second dose 11h after the infection (Theurl et al., 2011b; Poli et al., 2014a). Infection of mice was performed i.p. with either 1.1 x 10^6^ colony forming unites (CFU) of *E. coli* (O18:K1) or S.Tm (ATCC 14028), both suspended in 20 µl PBS, or PBS alone. Mice were terminated after 18h of infection and organ homogenates were plated in serial dilutions on Luria-Bertani (LB) agar (Sigma-Aldrich) plates to determine the bacterial load.

All animal experiments were performed in accordance with the Austrian Experimental Animal Welfare Act 2012 (Tierversuchsgesetz 2012) and were approved by the Federal Ministry of Science and Education (approval no. BMWFW-66.011/0115-V/3b/2019).

### RNA Extraction and Quantitative Real-Time PCR (qRT-PCR)

This was carried out as described previously (Dichtl et al., 2019). In brief, total RNA was prepared from liquid nitrogen-frozen mouse tissues using acid guanidinium thiocyanate-phenol-chloroform extraction with peqGOLD Tri-Fast™ (Peqlab). For reverse transcription 2 µg RNA, random hexamer primers (200 ng µl^-1^) (Roche), dNTPs (10 mM) (GE Healthcare LifeSciences) 20 U RNasin (Promega) and 200 U M-MLV reverse transcriptase (Invitrogen) in first strand buffer (Invitrogen) were used. TaqMan real-time PCR was performed on a CFX96 light cycler (Bio-Rad Laboratories GmbH). Ssofast Probes Supermix and Ssofast EvaGreen Supermix (Bio-Rad Laboratories GmbH) were used according to the manufacturer’s instructions. Real-time PCR reactions were performed on a CFX Cycler and analyzed with CFX software (Bio-Rad Laboratories GmbH). Gene expression was normalized using the ΔΔct method using *Rpl4* as reference transcript.

Primer sequences:

The following TaqMan PCR primers and probes were used (primer forward; primer reverse; probe):

Mouse *Rpl4*: 5’-CGCTGGTGGTGGAAGATAAGG-3’;
5’-CGGTTTCTCATTTTGCCCTTG-3’;
Cy5, 5’-CAGCCTCCTTGGTCTTCTTGTAACCTTC-3’, BHQ2
Mouse *Hamp*: 5’-GGCAGACATTGCGATACCAAT-3’;
5’-TGCAACAGATACCACACTGGGAA-3’;
FAM, 5’-CCAACTTCCCCATCTGCATCTTCTGC-3’
Mouse *Socs3*: 5’-GCGGGCACCTTTCTTATCC-3’;
5’-TCCCCGACTGGGTCTTGAC-3’;
FAM, 5’-AGCTCGGACCAGCGCCACTTCTTC-3’, BHQ1
Mouse *Kc*: 5’-CCGAAGTCATAGCCACACTCAA-3’;
5’-GCAGTCTGTCTTCTTTCTCCGTTAC-3’;
*Mouse Il6*: 5’-TGTTCTCTGGGAAATCGTGGA-3’;
5’-AAGTGCATCATCGTTGTTCATACA-3’;
FAM, 5’-ATGAGAAAAGAGTTGTGCAATGGCAATTCTG-3’, BHQ1
Mouse *Id1*: 5’-ATGTGTTCCAGCCGACG-3’;
5’-GGTAGTGTCTTTCCCAGAGATC-3’;
FAM, 5’-TCGCATCTTGTGTCGCTGAGGC-3’; BHQ1

### Plasma Measurements

Heparinized blood was centrifuged twice to obtain plasma. 50 µl of plasma were used for measurement of plasma iron using the QuantiChrome™ Iron Assay (BioAssay Systems) following the manufacturer’s protocol. ELISA kits were used to measure plasma concentrations of hepcidin [HMC-001 Hepcidin Murine-Compete ELISA Kit Intrinsic LifeSciences (ILS)], IL6 (BD OptEIA™) and LCN2 (Mouse Lipocalin-2/NGAL DuoSet ELISA; R&D Systems) following the manufacture’s protocol.

### Western Blot

Protein extraction and Western blotting were performed as described previously (Dichtl et al., 2019). The antibodies used were a rabbit Fpn1 antibody (1:2000; Eurogentec), a rabbit actin antibody (1:500; Sigma Aldrich), a rabbit Stat3 antibody (1:2000, Cell Signaling), a rabbit phospho-Stat3 antibody (1:2000, Cell Signaling), a rabbit phospho-Smad1/5/9 antibody (1:2000, Cell Signaling) and appropriate HRP-conjugated secondary antibodies (1:2000, anti-rabbit; Dako). For quantification, densitometry data were acquired on a ChemiDoc Touch Imaging System (Bio-Rad Laboratories GmbH) and analyzed with Quantity One software (Bio-Rad Laboratories GmbH).

### Statistics

Statistical analysis was carried out using GraphPad Prism version 8 for Windows (GraphPad Software). Data are presented as mean ± SD. Significant differences between groups were determined using a one-way ANOVA, two-way ANOVA (more than 2 groups, two factors) with Tukey-corrected post-hoc t-tests for multiple comparison and for only two groups a student t-test was performed. For non-normal distributed data, a Kruskal-Wallis test was performed. p < 0.05 was used as the significance threshold.

## Results

### LDN-193189 and Oversulfated Heparins Inhibit Hepcidin *In Vitro*

**Figure 1:**
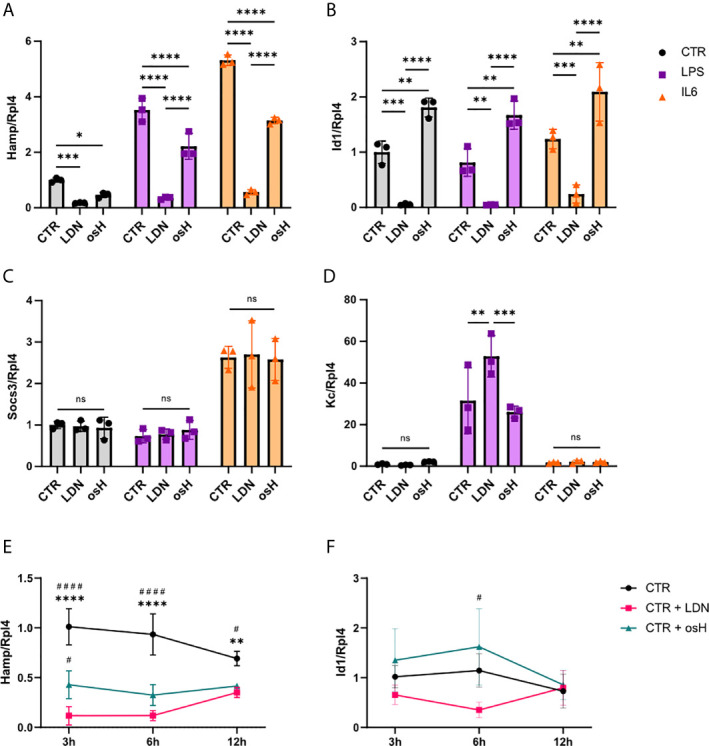
LDN-193189 and oversulfated heparins inhibit hepcidin expression in *vitro*. Relative mRNA expression of genes of interest in FL83B hepatocytes treated with various stimuli and inhibitors. Ribosomal Protein L4 (*Rpl4)* was used as a reference gene. Cells were incubated for 6h with either 500nM LDN-193189 (LDN) or with 3,6µg/ml of oversulfated heparins (osH). To simulate inflammatory conditions, cells were treated with 10ng/ml lipopolysaccharide (LPS) alone or together with one of the inhibitors or with 100ng/ml interleukin-6 (IL6) alone or together with one of the inhibitors. For this setup, the gene expression of **(A)** hepcidin antimicrobial peptide *(Hamp)* **(B)** Inhibitor of DNA binding 1 (*Id1)*, **(C)** suppressor of cytokine signaling 3 (*Socs3)* and **(D)** the murine homologue to IL8 (Kc) was determined. **(E)** Time course experiment with FL83B cells for *Hamp* expression and **(F)** *Id1* gene expression. **(A–F)** n = 3 per group. For **(A–D)**, only the differences between CTR, LDN and osH were calculated. For more than two groups a one-way ANOVA was performed. *p < 0.05, **p < 0.01, ***p < 0.001, ****p < 0.0001. ns, no significance of differences. For **(E, F)**, ^#^significant different from LDN treated cells, ^#^p < 0.05, ^####^p < 0.0001; *significant different from osH treated cells, **p < 0.01, ****p < 0.0001.

To determine if LDN-193189 (LDN) or over-sulfated heparins (osH) can inhibit *Hamp* expression *in vitro*, we started experiments with murine FL83B cells, which are immortalized hepatocytes (Breslow et al., 1973).When unstimulated cells were treated with either LDN or osH for 6h, we observed a significant reduction of *Hamp* expression as determined by qPCR with LDN being more potent than osH at the dosages used (**Figure 1A**). In addition, we wanted to know if the two substances can inhibit *Hamp* expression under inflammatory conditions. Therefore, we treated FL83B hepatocytes with either LPS or IL6 to provoke an inflammatory stimulus and added LDN or osH for 6h to inhibit *Hamp* expression. LPS and IL6 increased *Hamp* expression as expected, while concomitant addition of LDN and osH reduced *Hamp* expression significantly, whereby LDN had a stronger effect in both settings (**Figure 1A**). To determine which signaling pathway may be inhibited we first had a look at the mRNA expression of the inhibitor of DNA binding 1 (*Id1*). Like *Hamp*, *Id1* is a target gene of the BMP-SMAD pathway and thereby an indicator for an activation of this signaling cascade (Miyazono and Miyazawa, 2002). *Id1* expression was neither induced by LPS- nor IL6-treatment but strongly decreased after the addition of LDN (**Figure 1B**). In contrast, the addition of osH to the untreated, LPS- or IL6-treated cells did not reduce *Id1* expression but rather increased it (**Figure 1B**). Suppressor of cytokine signaling 3 (SOCS3) is known as a negative feedback regulator of cytokine signaling through the Jak/STAT pathway and is an indicator that the inflammatory IL6-driven pathway is active (Miyachi et al., 2011). *Socs3* gene expression was upregulated by IL6 but not upon LPS stimulation (**Figure 1C**). LPS can activate *Hamp* expression *via* TLR4 –MyD88 signaling. Therefore, we examined the gene expression of the murine homologue to human IL8 named *Kc* which is a target gene of this pathway. Gene expression of *Kc* was upregulated following LPS treatment and the inhibitors did not reduce its expression. The treatment with IL6 did not significantly affect *Kc* expression (**Figure 1D**). To get a better overview of the kinetics of *Hamp* suppression by the two inhibitors, we performed a time course experiment. LDN and osH effectively inhibited *Hamp* at 3h, 6h and 12h, though the effect declined between 6h and 12h (**Figure 1E**). Interestingly, *Id1* expression did not parallel *Hamp* expression, and differences between LDN and osH were only observed at 6h (**Figure 1F**).

Next, we evaluated if the solvents (vehicles) used, i.e., 2% (wt/vol) (2-Hydroxypropyl)-β-cyclodextrin (CD) for LDN and PBS for osH, may influence the different pathways. To this end, we evaluated the mRNA expression of various genes involved in the different pathways under both control (CTR) and LPS- or IL6-treatment conditions, respectively (**Supplementary Figures 1A–D**). Further, no changes in *Il6* gene expression were observed due to the treatment with those vehicles (**Supplementary Figure 1E**). Therefore, the vehicles alone do not influence hepcidin signaling and do not seem to have a pro- or anti-inflammatory effect.

Thus, LDN and osH are selective and competent hepcidin inhibitors *in vitro*. Both can even act as hepcidin suppressors in an inflammatory setup, but do not generally impair the inflammatory signals per se.

### LDN-193189 and Oversulfated Heparins Inhibit Hepcidin *In Vivo* in Uninfected Mice but Not in Mice With Systemic Bacterial Infection

**Figure 2:**
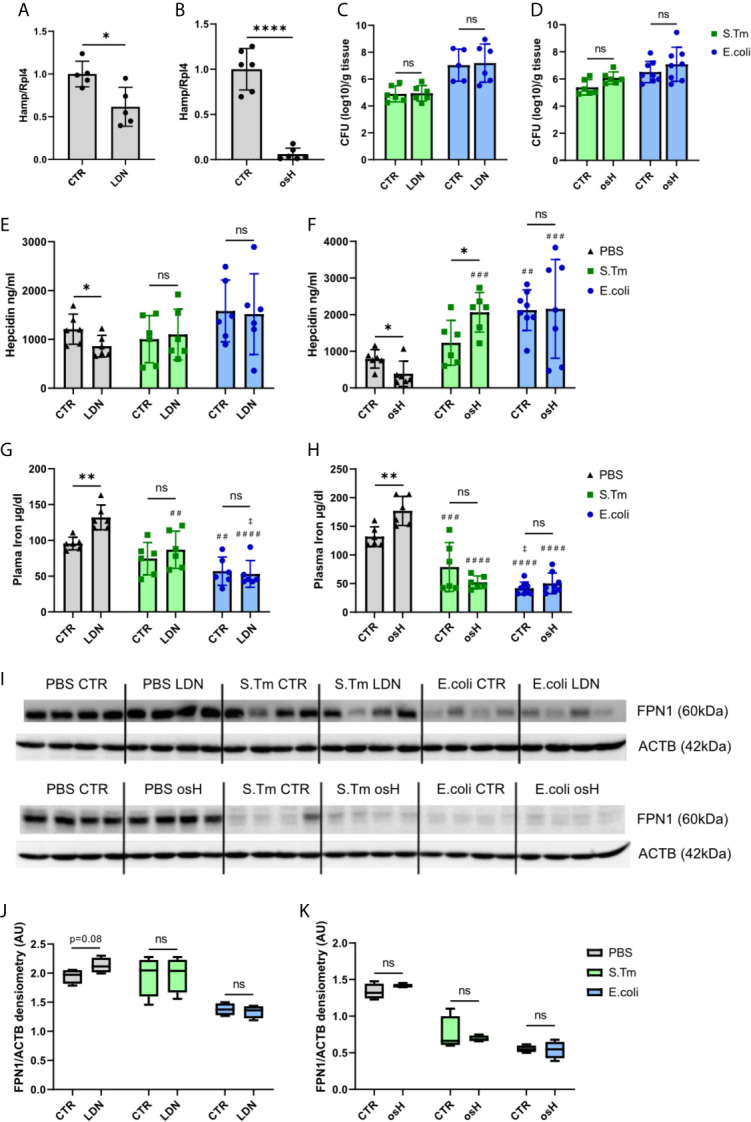
LDN-193189 and oversulfated heparins inhibit hepcidin in *vivo* in uninfected mice but no in infected mice. Ten weeks old, male, C57BL/6N mice were treated with **(A)** 3mg/kg-bodyweight LDN-193189 (LDN) or **(B)** 40mg/kg-bodyweight of oversulfated heparins (osH) for 18h. The mice received an injection of the inhibitor or the vehicle (CTR) at time point 1h prior to infection and a second dose11h after infection. Mice were infected with 1.1 x 10^6^ colony forming units (CFU) of either *Salmonella* Typhimurium (S.Tm), *Escherichia coli* (*E.coli*) or just PBS as a control. Splenic bacterial load of mice receiving **(C)** LDN or **(D)** osH. Plasma hepcidin of mice receiving **(E)** LDN or **(F)** osH was measured using an ELISA. Plasma iron of mice receiving **(G)** LDN or **(H)** osH. **(I)** Protein levels of splenic ferroportin (FPN1) and actin (ACTB) as a reference of infected mice receiving the two different hepcidin inhibitors and **(J, K)** the quantification of the Western blot results. Each dot indicates a single mouse. **(A, B)** n=5 per group, **(C–H)** n=5-8 per group. One statistical outlier was excluded in **(F)** for E.coli/osH. For **(I)** 4 representative samples of each group were chosen. A two-way ANOVA was performed where more than 2 groups existed and a student t-test was performed for results with only 2 groups. *p < 0.05, **p < 0.01, ****p < 0.0001, ns, no significance of differences; ^#^significantly different from uninfected mice, ^##^p<0.01, ^###^p < 0.001, ^####^p < 0.0001; ‡ significantly different from S.Tm infected mice, ^‡^p<0.05.

As both hepcidin inhibitors efficiently inhibited *Hamp* mRNA *in vitro*, we further investigated their potential for blocking *Hamp* expression *in vivo.* Employing male C57BL/6N mice, both inhibitors were, given twice, i.e., 19 hours and 7 hours before sacrifice. Notably, both compounds were able to reduce the expression of hepatic *Hamp*, with osH being a more efficient inhibitor than LDN at the indicated time point (**Figures 2A, B**). As both inhibitors were able to reduce *Hamp* expression *in vitro* in LPS treated cells, we then assessed their effects on *Hamp* in an infection model *in vivo* using two Gram-negative bacteria, either S.Tm, an intercellular pathogen, or *E.coli*, an extracellular pathogen, either of which was injected intraperitoneally (i.p.). Mice received two injections of either LDN or osH, i.e., the first 1h prior and the second 11h after the infection. After a total of 18h of infection, mice were sacrificed. Of interest, bacterial numbers in the spleen did not show any significant differences when comparing inhibitor treated mice with solvent treated mice for each of the pathogens (**Figures 2C, D**). In uninfected mice, plasma hepcidin levels were reduced following administration of either inhibitor (**Figures 2E, F**). In S.Tm infected mice, the inhibitors did not reduce hepcidin levels, while in osH treated mice, hepcidin was even increased (**Figure 2F**). *E.coli* infected mice had high levels of hepcidin, while LDN and osH were unable to inhibit hepcidin formation (**Figures 2E, F**). High hepcidin levels reduce plasma iron concentration by binding of hepcidin to the iron exporter ferroportin (FPN1), thereby reducing the iron export from cells into the plasma. Therefore, we measured plasma iron concentrations and as expected, uninfected mice, in which the inhibitor reduced hepcidin levels, had increased plasma iron concentrations (**Figures 2G, H**). However, we could not detect differences of plasma iron levels upon treatment with the hepcidin inhibitors following infection with either S.Tm or *E.coli* (**Figures 2G, H**). We then studied for a possible effect of hepcidin -inhibiting treatment towards FPN1 protein expression in the spleen. Of note, we could not find differences between untreated and mice treated with the inhibitors throughout all the groups (**Figures 2I–K**). Uninfected mice, treated with either of the inhibitors (LDN or osH) had a slightly increased FPN1 expression, which did not reach significance at the investigated time point (**Figures 2I–K**). Splenic *Fpn* mRNA expression did not differ between inhibitor treatment and vehicle treatment but was decreased in *E.coli* infected mice, which may be due to increased concentrations of pro-inflammatory stimuli including LPS and IL6, a mechanism that had been previously described (Ludwiczek et al., 2003; Liu et al., 2005; Guida et al., 2015) (**Supplementary Figures 1F, G**). These data suggest that both inhibitors can inhibit *Hamp* expression in uninfected mice resulting in increased plasma iron levels but appear to be ineffective to block hepcidin expression in mice suffering from systemic bacterial infection.

### Hepcidin Signaling in Infected and Uninfected Mice and Upon Hepcidin Signaling Inhibition

**Figure 3:**
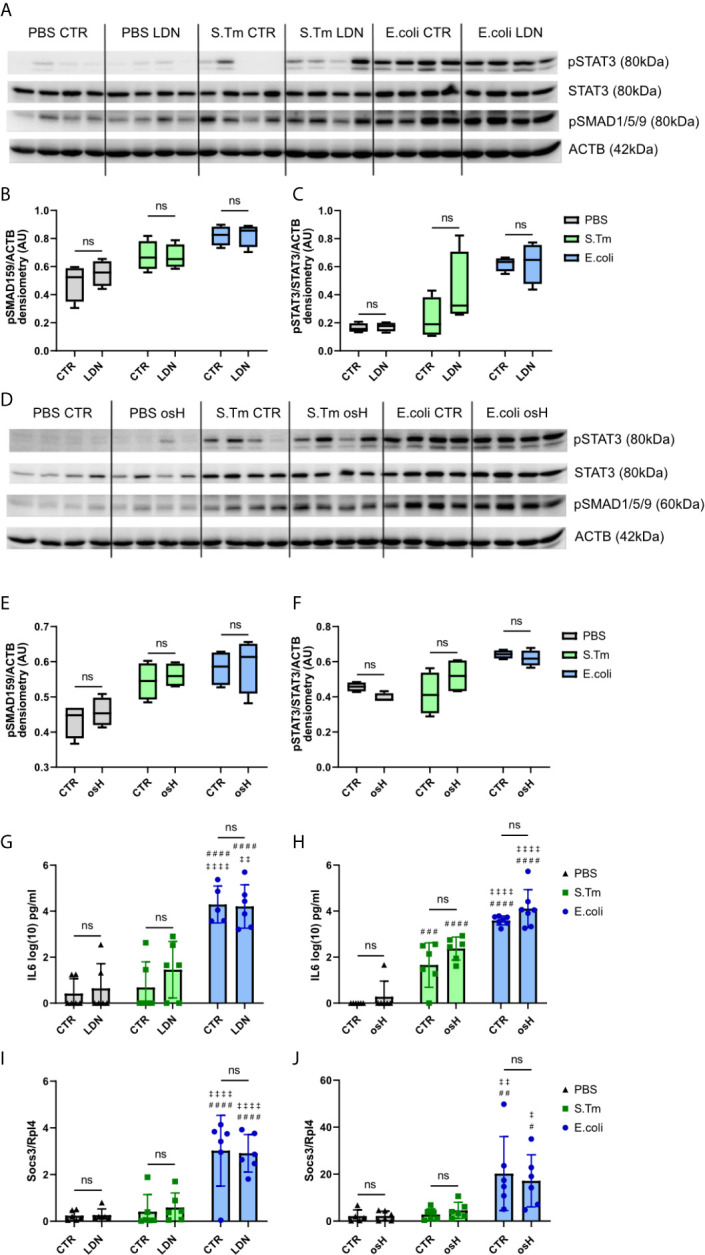
Hepcidin regulation. **(A, D)** Western blots of liver protein extracts of phosphorylated signal transducer and activator of transcription–3 (pSTAT3), STAT3, phosphorylated SMA and mothers against decapentaplegic-1/5/9 (pSMAD1/5/9) and actin (ACTB) of infected mice receiving the different hepcidin inhibitors are shown. **(B, C, E, F)** Quantification of the Western blot results. **(G, H)** Interleukin-6 (IL6) plasma levels in log(10) pg/ml measured using an ELISA kit. **(I, J)** Realtime PCR results of hepatic gene expression of suppressor of cytokine signaling-3 (*Socs3)* with Ribosomal Protein L4 (*Rpl4)* as a reference receiving either LDN-193189 (LDN), oversulfated heparins (osH) or the respective vehicle (CTR). For **(A, D)**, 4 representative samples of each group were chosen. **(G–J)** each dot indicates a single mouse. **(G–J)** n = 5-7 per group. A two-way ANOVA was performed for the results with more than 2 groups. ns: no significance of differences**;** ^#^significantly different from uninfected mice, ^##^p < 0.01, ^###^p < 0.001, ^####^p < 0.0001; ‡ significantly different from S.Tm infected mice, ^‡^p < 0.05, ^‡‡^p < 0.01, ^‡‡‡‡^p < 0.0001.

We next studied hepcidin signaling pathways using Western blot analysis for phosphorylated signaling proteins. The results showed that the BMP-SMAD pathway is induced by either *E.coli* or S.Tm infection, as reflected by phosphorylation of SMAD1/5/9 (**Figures 3A, B, D, E**). However, neither LDN nor osH impaired bacteria induced increased phosphorylation. *Id1* is a target gene of the BMP-SMAD pathway, but did not show any differences between the two inhibitors in infected and uninfected mice (**Supplementary Figures 1H, I**). Phosphorylated STAT3 is a part of the IL6-STAT3 inflammatory pathway and STAT3 phosphorylation is increased upon infection with S.Tm and *E.coli but* is not affected by either osH or LDN (**Figures 3A, C, D, F**). Since IL6 is an activator of the inflammatory pathway, we measured plasma levels of this cytokine, but could not find differences between LDN and osH treated mice as compared to infected mice without hepcidin inhibitor treatment (**Figures 3G, H**). As IL6 levels are low for S.Tm infected mice, we also determined plasma Lipocalin-2 (LCN2), as an acute phase protein produced upon infection with Gram-negative bacteria (Chakraborty et al., 2012; Nairz et al., 2015b). LCN2 did not differ between vehicle and inhibitor treatment and was significantly higher in S.Tm and *E.coli* mice being highest in the latter (**Supplementary Figures 1H, I**). Accordingly, LDN and osH did not change the *Socs3* expression of uninfected, S.Tm or *E.coli* infected mice (**Figures 3I, J**).

## Discussion

Hepcidin plays a central role in the orchestration of iron homeostasis and the alterations of iron trafficking during infection, both on a systemic and cellular level (Pagani et al., 2011; Ganz and Nemeth, 2015; Haschka et al., 2020). Specifically, spatio-temporal changes in cellular iron levels promote either the growth of intracellular or extracellular microbes (Drakesmith and Prentice, 2012; Nairz et al., 2014). Therefore, targeting hepcidin expression could be a promising target for specific infections (Arezes et al., 2015; Nairz et al., 2015a). In this study, we used two different hepcidin inhibitors, that were already shown to be effective *in vitro* and *in vivo*, to evaluate their impact on the course of infections with intracellular versus extracellular bacteria. LDN-193189, an inhibitor of the type I BMP receptors ALK2 and ALK3 signaling pathways and oversulfated heparins, which bind to BMPs, thereby blocking the signaling induction *via* BMP6, were able to reduce *Hamp* transcription *in vitro.* Of note *Id1* expression was not inhibited by osH *in vitro*, what is in contrast to previous findings (Poli et al., 2014a). The fact that *Id1* mRNA was not affected by osH, whereas *Hamp* was, raises the question, which pathway is inhibited by osH. *In vivo*, healthy mice responded to both inhibitors with decreased hepcidin levels and thus increased plasma iron levels. Beside the inhibition of hepcidin, phospho-SMAD1/5/9 was detectable and no differences were seen between the inhibitors and control groups after 18h. A possible explanation is that the influence of the inhibitors on the pathway is an immediate and transient effect and cannot be detected after a longer time period (Wang et al., 2005; Casanovas et al., 2014; Camaschella et al., 2020), as in our case after 18h. The transient effect of osH *in vivo* was displayed by Poli et al. where they showed that hepatic hepcidin mRNA expression reached its minimum after 3h and returned to baseline after 12 hours with changes in serum hepcidin levels being a little delayed (Poli et al., 2014a). However, none of the inhibitors showed an effect in infected mice on hepcidin expression, its circulating levels or on serum iron concentrations.

Our results indicated that both pathways, the IL6-STAT3 and the BMP-SMAD, are activated upon *Salmonella* infection and even more during *E.coli* infection which translated into higher circulating hepcidin levels in the latter. Neither of the inhibitors showed an effect on the two *Hamp* signaling pathways in infected animals at the time point investigated, and plasma levels of hepcidin were not reduced with these two inhibitors. As both inhibitors did not decrease hepcidin plasma levels nor impacted on ferroportin expression in the spleen, it was not unexpected that the CFU of both bacterial species investigated in the spleens did not differ with or without *Hamp* signaling inhibitor treatment.

As the two inhibitors with their proposed effects on SMAD1/5/9 signaling showed neither an inhibitor effect nor altered STAT3 phosphorylation, other hepcidin inducing pathways may play a dominant role during bacterial infection (Girelli et al., 2016; Nairz and Weiss, 2020).

The hepcidin signaling contains different pathways and there is evidence that they can crosstalk and have a certain dominance over each other depending on iron availability, hypoxic response, or inflammatory activity (Theurl et al., 2011a; Girelli et al., 2016). An indication is the proximity between the STAT3 responsive element (STAT3-RE), which is the target of the IL6/STAT3 pathway and the BMP responsive element (BMP-RE) of the BMP-SMAD pathway, in the *Hamp* promotor. Thus, inactivation of the BMP-RE also decreases IL6 induced *Hamp* activation (Casanovas et al., 2009). Accordingly, *Hamp* is prominently induced *via* the ALK2/ALK3 pathway and its inhibition can suppress hepcidin expression (Steinbicker et al., 2011a; Mayeur et al., 2014; Asshoff et al., 2017; Belot et al., 2019). Further, a liver conditional knockout of either *Alk3* or *Smad4* inhibits *Hamp* expression in response to LPS and IL6 (Wang et al., 2005; Mayeur et al., 2014). Activin B was discussed to be a potential regulator in the crosstalk between the inflammatory and the iron dependent pathway for *Hamp* expression, as it led to inflammation driven *Hamp* induction independent of the SAMD-BMP pathway, but did not meet expectations (Besson-Fournier et al., 2012; Besson-Fournier et al., 2017). MyD88 is part of the TLR4 hepcidin-signaling induced by LPS (Lee et al., 2017) and is required for appropriate *Hamp* expression in response to dietary iron in mice (Layoun et al., 2018). Consequently, MyD88 mediated signaling could be central for hepcidin induction upon bacterial infection *in vivo* and is not properly targeted by the inhibitors used. Conversely, due to the vital role of hepcidin in host response to infection and being aware of the multiple signals which regulate hepcidin expression, it is likely that inhibition of one or two signals will result in compensatory stimulation of salvage signaling pathways to ensure proper hepcidin formation during host response against infection (Ganz and Nemeth, 2015; Girelli et al., 2016; Nairz and Weiss, 2020). Subsequent studies will have to clarify how combinations of hepcidin inhibitors which also target those signaling pathways for hepcidin induction will impact on the outcome of infectious disease and clarify the role of hepcidin for host control of infection with intracellular or extracellular microbes.

Anemia of inflammation (AI) occurs in the setting of acute and chronic inflammatory diseases including infections (Weiss et al., 2019). One of its pathophysiologic hallmarks is the sequestration of iron in intracellular compartments, which is pivotally driven by hepcidin. Therefore, blocking *Ham*p transcription is a promising approach for the therapy of AI and predicted to increase the availability of iron for erythropoiesis in the bone marrow (Petzer et al., 2018). Correspondingly, hepcidin inhibitors are being evaluated in clinical trials for their efficacy to improve AI and related entities such as the anemia in chronic kidney disease or the anemia of the elderly (Stauder et al., 2018). As these forms of anemia affect patients at increased risk of infections, the safety profile of compounds targeting *Hamp* transcription needs to be thoroughly assessed, as iron released from macrophages may be easily accessible to circulating microbes. The latter has been shown to be of concern in patients with impaired immune response and high iron availability, as in sepsis patients or patients after allogenic bone marrow transplantation (Petzer et al., 2019; Brandtner et al., 2020).

There are limitations in this study. According to the study design, we were only able to investigate the effects of the different inhibitors at a single time point, namely at 18h after infection. For subsequent studies, earlier timepoints could be analyzed to have a better overview on the dynamic effects of those inhibitors on hepcidin expression. Since in our setup of *in vitro* experiments both inhibitors were capable to reduce *Hamp* expression with to LPS or IL6 simulation, and osH did not diminish *Id1* mRNA in any of the setups, the precise mechanism of inhibition and the affected pathways need further investigations. Our focus was to evaluate the systemic iron homeostasis on bacterial numbers. Further studies should focus on the crosstalk of systemic iron regulation pathways, immune mechanisms and specific pathogens, and how putative compensation mechanisms can circumvent inhibition of BMP-SMAD expression during infection. Such analyses may lead to identification of new pathways in hepcidin regulation and may uncover potential targets for a successful inhibition of hepcidin formation in infections. Despite these limitations, our data showed that the two hepcidin inhibitors worked well *in vitro* under inflammatory conditions and also *in vivo* in uninfected mice but they could not inhibit *Hamp* expression *in vivo* upon infections with *E.coli* and S.Tm. Thus, we could neither find systemic effects in iron homeostasis nor differences in bacterial loads in organs of infected mice as a function of hepcidin inhibitor treatment. This suggests that, *in vivo*, multiple factors can influence hepcidin expression and compensate the inhibition of one major signaling pathway.

## Data Availability Statement

The raw data supporting the conclusions of this article will be made available by the authors, without undue reservation.

## Ethics Statement

The animal study was reviewed and approved by Austrian Experimental Animal Welfare Act 2012 Federal Ministry of Science and Education.

## Author Contributions

AH and GW planed and designed the project. AH, LVS, DH, MS, LR, MP, PG, MG and AM performed or helped with experiments. AH did the visualization of the data and performed the statistical analysis. AH, DH, MN and GW prepared and created the initial draft. LVS, MN, DH, LR, PG, MG, AM and GW were included in the critical review and writing of the manuscript. GW was responsible for supervision and funding acquisition. All authors contributed to the article and approved the submitted version.

## Funding

Financial support by the Christian Doppler Laboratory of iron metabolism and anemia research is gratefully acknowledged. This work was supported by the ‘Verein zur Förderung von Forschung und Weiterbildung in Infektiologie und Immunologie an der Medizinischen Universität Innsbruck’ and by funds of the Austrian Science Fund (FWF W1253-B24; doctoral program HOROS).

## Conflict of Interest

The authors declare that the research was conducted in the absence of any commercial or financial relationships that could be construed as a potential conflict of interest.

## Publisher’s Note

All claims expressed in this article are solely those of the authors and do not necessarily represent those of their affiliated organizations, or those of the publisher, the editors and the reviewers. Any product that may be evaluated in this article, or claim that may be made by its manufacturer, is not guaranteed or endorsed by the publisher.

## Supplementary Material

Supplementary Figure 1 FL83B cells were left untreated (CTR), treated with 2% (wt/vol) (2-Hydroxypropyl)-β-cyclodextrin (CD) or PBS to investigate the influence of the vehicles of the inhibitors on the different pathways. In addition, cells were left untreated or stimulated with lipopolysaccharide (LPS) or interleukin-6 (IL6) to evaluate the gene expression of **(A)** hepcidin (*Hamp*), **(B)** inhibitor of DNA binding (*Id1*), **(C)** suppressor of cytokine signaling 3 (*Socs3)*, **(E)** murine homologue to IL8 *(Kc)* and interleukin-6 (*Il6*). Ten weeks old, male C57BL/6N mice were treated with 3mg/kg-bodyweight LDN-193189 (LDN) or 40mg/kg-bodyweight of oversulfated heparins (osH) for 18h. The mice received an injection of the inhibitor or the vehicle (CTR) at time point 1h prior to infection and a second dose 11h after the infection. Mice were infected with 1.1 x 10^6^ colony forming units of either *Salmonella* Typhimurium (S.Tm), *Escherichia coli* (*E.coli*) or received PBS as a control. **(F, G)** Gene expression of splenic ferroportin (Fpn). **(H, I)** Plasma lipocalin 2 (LCN2) levels and **(J, K)** hepatic gene expression of *Id1*Ribosomal Protein L4 (*Rpl4)* was used as a reference gene for qPCR. For **(A–E)** n=3 per group. **(F–K)** each dot indicates a single mouse. n= 5-8 per group. A two-way ANOVA was performed for the results with more than 2 groups. **(A–E)**: Differences between the group of untreated (CTR), LPS, and IL6 treated cells are shown with a #: ^##^p < 0.01, ^###^p < 0.001, ^####^For **(F–K)**: ^#^significantly different from uninfected mice, ^#^p < 0.05, ^###^p < 0.001, ^####^p < 0.0001; ^‡^significantly different from S.Tm infected mice, ^‡^p < 0.05, ^‡‡^p < 0.01, ^‡‡‡‡^p < 0.0001. ns, no significance of differences.

## Abbreviations

µg, microgram; ALK, activin receptor like kinase; AP-1, activator protein-1; BMP, bone morphogenic protein; Cxcl8, CXC-motiv-chemokin 8; DMT1, divalent metal transporter; E.coli, Escherichia coli; Figure, figure; FPN1, ferroportin; h, hour; Hamp, hepcidin; i.p., intra peritoneal; Id1, inhibitor of DNA binding; IL6, interleukin 6; IRAK, interleukin- receptor-associated kinase; JAK2, janus kinase 2; JNK, c-Jun N-terminal kinase; kg, kilogram; LCN2, Lipocalin 2; LDN, LDN-193189; LPS, lipopolysaccharide; ml, milliliter; mM, millimolar; MyD88, myeloid differentiation primary response 88; ng, nanogram; nM, nanomolar; Nramp1, natural resistance-associated macrophage protein 1; osH, oversulfated Heparins; qPCR, qualitative real time polymerase chain reaction; RE, response element; S.Tm, Salmonella Typhimurium; SCV, Salmonella containing vacuole; SMAD, SMA and mothers against decapentaplegic; SOCS3, suppressor of cytokine signaling 3; STAT3, signal transducer and activator of transcription – 3; TLR4, toll like receptor 4; TRAF6, tumor necrosis factor receptor-associated factor 6; VH, vehicle; Zip14, solute carrier family 39 (zinc transporter) member 14.
