# Supplementary figures and images for: Pharmacological Targeting of BMP6-SMAD Mediated Hepcidin Expression Does Not Improve the Outcome of Systemic Infections With Intra-Or Extracellular Gram-Negative Bacteria in Mice
Source: Front Cell Infect Microbiol. 2021 Jul 23;11:705087. doi: 10.3389/fcimb.2021.705087 (PMC8342937; doi:10.3389/fcimb.2021.705087)

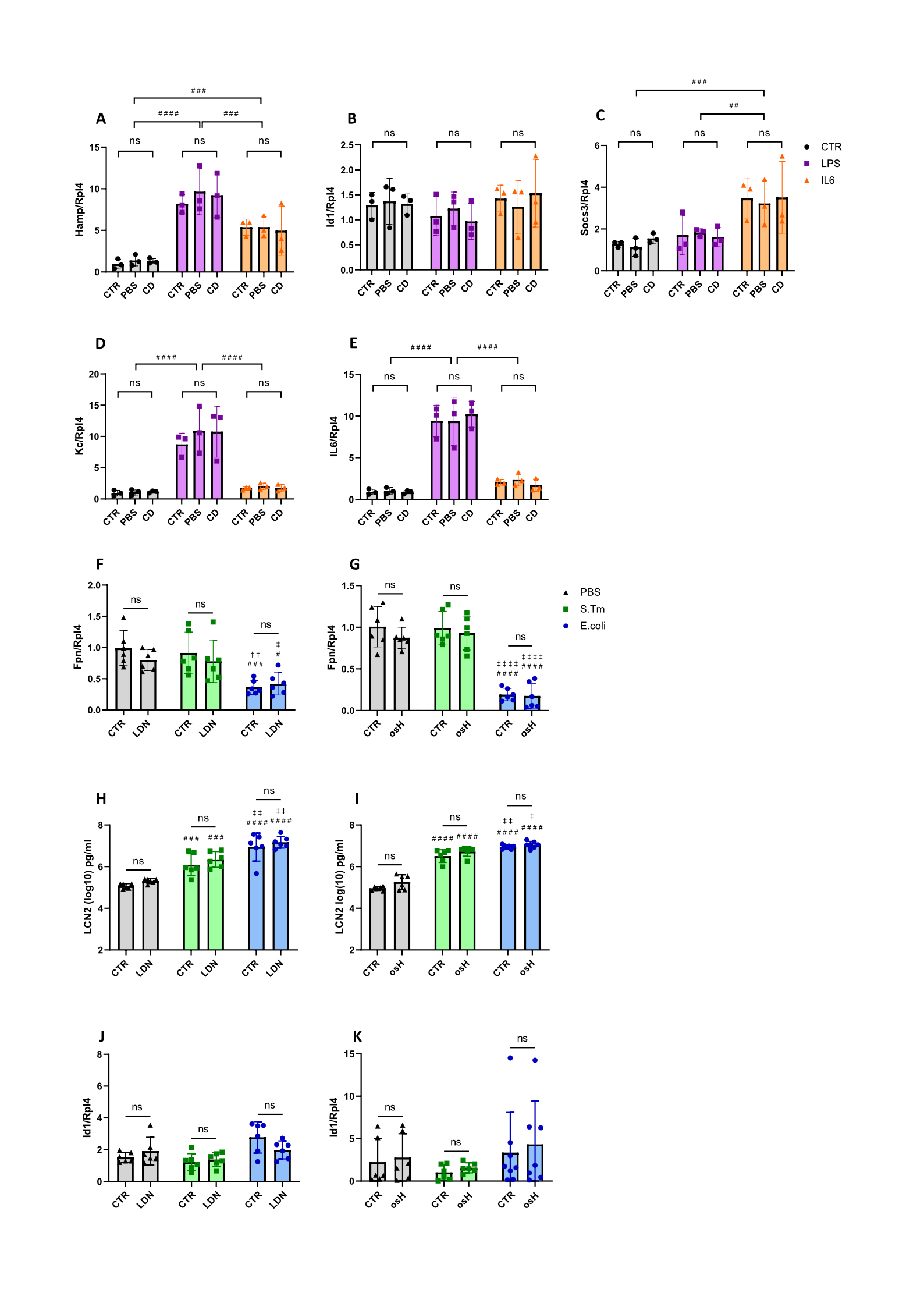

Supplement: Supplementary Figure 1 — FL83B cells were left untreated (CTR), treated with 2% (wt/vol) (2-Hydroxypropyl)-β-cyclodextrin (CD) or PBS to investigate the influence of the vehicles of the inhibitors on the different pathways. In addition, cells were left untreated or stimulated with lipopolysaccharide (LPS) or interleukin-6 (IL6) to evaluate the gene expression of (A) hepcidin (Hamp), (B) inhibitor of DNA binding (Id1), (C) suppressor of cytokine signaling 3 (Socs3), (E) murine homologue to IL8 (Kc) and interleukin-6 (Il6). Ten weeks old, male C57BL/6N mice were treated with 3mg/kg-bodyweight LDN-193189 (LDN) or 40mg/kg-bodyweight of oversulfated heparins (osH) for 18h. The mice received an injection of the inhibitor or the vehicle (CTR) at time point 1h prior to infection and a second dose 11h after the infection. Mice were infected with 1.1 x 10^6^ colony forming units of either Salmonella Typhimurium (S.Tm), Escherichia coli (E.coli) or received PBS as a control. (F, G) Gene expression of splenic ferroportin (Fpn). (H, I) Plasma lipocalin 2 (LCN2) levels and (J, K) hepatic gene expression of Id1Ribosomal Protein L4 (Rpl4) was used as a reference gene for qPCR. For (A–E) n=3 per group. (F–K) each dot indicates a single mouse. n= 5-8 per group. A two-way ANOVA was performed for the results with more than 2 groups. (A–E): Differences between the group of untreated (CTR), LPS, and IL6 treated cells are shown with a #: ##p < 0.01, ###p < 0.001, ####For (F–K): #significantly different from uninfected mice, #p < 0.05, ###p < 0.001, ####p < 0.0001; ‡significantly different from S.Tm infected mice, ‡p < 0.05, ‡‡p < 0.01, ‡‡‡‡p < 0.0001. ns, no significance of differences. [file Image_1.tiff]
